# Fasting, non-fasting and postprandial triglycerides for screening cardiometabolic risk

**DOI:** 10.1017/jns.2021.73

**Published:** 2021-09-14

**Authors:** Bryant H. Keirns, Christina M. Sciarrillo, Nicholas A. Koemel, Sam R. Emerson

**Affiliations:** 1 Department of Nutritional Sciences, Oklahoma State University, Stillwater, OK74075, USA; 2 Boden Collaboration for Obesity, Nutrition, Exercise and Eating Disorders, The University of Sydney, Sydney, NSW2006, Australia; 3 Sydney Medical School, The University of Sydney, Sydney, NSW2006, Australia

**Keywords:** Cardiometabolic, Cardiovascular disease, Fasting triglycerides, Non-fasting triglycerides, Postprandial triglycerides, Risk screening, Triglyceride-rich lipoproteins

## Abstract

Fasting triacylglycerols have long been associated with cardiovascular disease (CVD) and other cardiometabolic conditions. Evidence suggests that non-fasting triglycerides (i.e. measured within 8 h of eating) better predict CVD than fasting triglycerides, which has led several organisations to recommend non-fasting lipid panels as the new clinical standard. However, unstandardised assessment protocols associated with non-fasting triglyceride measurement may lead to misclassification, with at-risk individuals being overlooked. A third type of triglyceride assessment, postprandial testing, is more controlled, yet historically has been difficult to implement due to the time and effort required to execute it. Here, we review differences in assessment, the underlying physiology and the pathophysiological relevance of elevated fasting, non-fasting and postprandial triglycerides. We also present data suggesting that there may be a distinct advantage of postprandial triglycerides, even over non-fasting triglycerides, for early detection of CVD risk and offer suggestions to make postprandial protocols more clinically feasible.

## Graphical abstract

**Figure:**
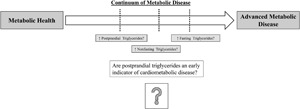

## Introduction

High fasting triglycerides have been associated with cardiovascular disease (CVD) since the 1950s, and nearly one-third of Americans display elevated triglycerides (>1⋅70 mmol/l or 150 mg/dl)^(1,2)^. Triglycerides >1⋅70 mmol/l (150 mg/dl) are also one criterion for diagnosis of the metabolic syndrome and are frequently observed in those with type 2 diabetes^(3–6)^. Chronically elevated fasting triglycerides in the absence of a genetic lipid disorder are associated with some degree of metabolic derangement, which can include long-term positive energy balance and adipose tissue expansion, hepatic steatosis and subsequent very-low-density lipoprotein (VLDL) oversecretion and/or insulin resistance^(1,7–16)^. In addition to their relationship with these metabolic changes, fasting triglycerides strongly correlate with other adverse, and more complex, lipid profile changes that are less easily measured (e.g. increased small, dense low-density lipoprotein (LDL), increased LDL triglycerides)^(17,18)^. Given that 84 % of those with fasting triglycerides marginally above the recommended cut-off (i.e. >2⋅0 mmol/l or 177 mg/dl) with increased waist circumference also display elevated insulin, apolipoprotein (apo)B and small LDL-particles , fasting triglycerides are likely most useful in screening for more moderate stages of declining metabolic health^(19)^.

**Fig. 1.**
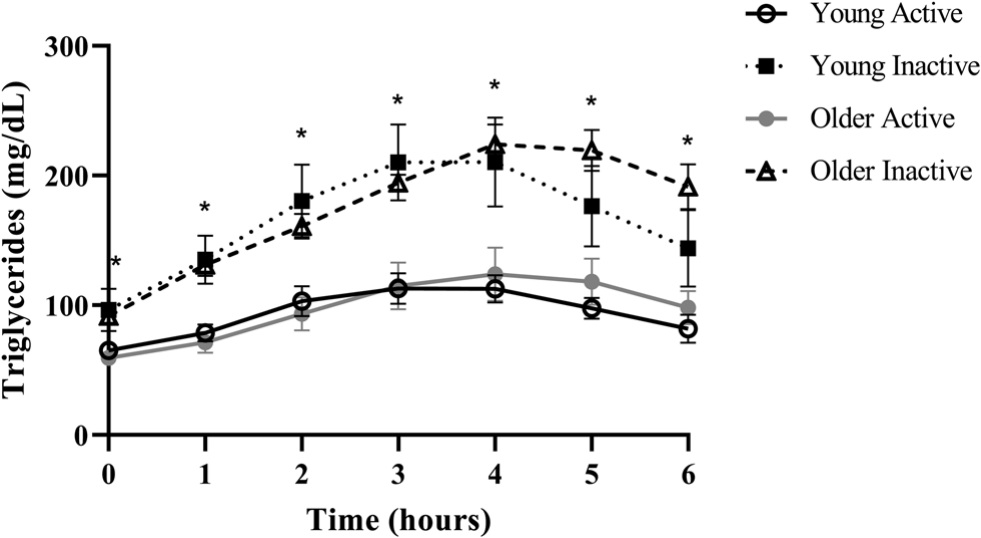
Postprandial triglycerides in active and inactive individuals with healthy fasting triglycerides. Despite normal fasting triglycerides, glucose, and HDL, disease-free, inactive individuals experienced an adverse postprandial triglyceride response. Young adults were 18–35 years old and older adults were ≥60 years. Physically active was defined as ≥150 min moderate to vigorous physical activity per week and physically inactive was <150 min moderate to vigorous physical activity per week and <30 min planned exercise per week. Significant differences (denoted by *; *P* < 0⋅05) were observed at every time point when comparing pooled inactive *v.* active individuals using Bonferroni corrected independent *t*-tests. Data reproduced with permission.

Despite triglycerides historically being measured in the fasted state, the rise in triglycerides following a meal, or postprandial lipaemia, has become increasingly examined due to epidemiological evidence that non-fasting triglycerides (i.e. triglycerides measured within 8 h of an unstandardised meal) are a strong predictor of CVD, and in some cases, more closely related to risk than fasting triglycerides^(20–22)^. A recent report from our group in generally healthy younger (18–35 years) and older (≥60 years) individuals defined as either active (i.e. ≥150 min moderate to vigorous physical activity/week) or sedentary (i.e. <150 min moderate to vigorous physical activity/week and <30 min planned exercise/week) also illustrates this point using a postprandial triglyceride assessment (serial measurement of triglycerides after a standardised test meal). Inactive participants presented with normal fasting triglycerides (1⋅10 mmol/l or 97⋅4 mg/dl), glucose and high-density lipoprotein cholesterol (HDL-C), but following a high-fat meal challenge (Marie Callendar's chocolate pie; 12 kcal/kg; 63 % fat (47 % saturated fat)) experienced a 236 % increase in peak triglycerides (2⋅60 mmol or 230⋅3 mg/dl) and a large triglyceride area under the curve relative to active counterparts (Fig. 1)^(23)^. Importantly, this increase in postprandial triglycerides is above the current recommended threshold for what is considered adverse (i.e. ≥2⋅48 mmol/l or 220 mg/dl), and in this population, was one of the only apparent signs of declining metabolic health^(22,24)^. Therefore, a large postprandial lipemic response may serve as a sensitive risk detection tool in apparently healthy individuals. This exaggerated triglyceride response could be due to many factors including intestinal and hepatic triglyceride-rich lipoprotein (TRL) oversecretion, reduced LPL activity and LPL saturation^(1,25–34)^. TRL remnants (i.e. VLDL and chylomicrons with partially hydrolysed triglyceride cargo) appear to account for the increased CVD risk in those with high post-meal triglycerides because they are selectively retained in the subintimal space, readily engulfed by macrophages in their unmodified form, and contain 5–20× the cholesterol content of LDL, making them highly atherogenic^(18,35–38)^. Overall, a growing body of epidemiological and clinical evidence suggests that the rise in triglycerides after a meal may be a more sensitive screening tool than fasting triglycerides for detecting disease risk and may be abnormal when other traditional risk factors (i.e. fasting triglycerides, HDL-C, glucose) are in the normal range.

Given the usefulness of post-meal triglyceride measurement for predicting CVD, it is important to further delineate between non-fasting and postprandial triglyceride assessment, which are often described interchangeably. Measurement of non-fasting triglycerides is one such method and describes a triglyceride measurement taken anytime within 8 h of eating a free-living, unstandardised meal^(22)^. Given this wide assessment window, non-fasting triglycerides are conveniently measured and often used in large epidemiological studies, but usually cannot control for a variety of factors that modify triglycerides (e.g. meal composition and timing, recent physical activity) and there is a possibility that peak post-meal triglycerides are not captured. The other form of triglyceride assessment after a meal is known as postprandial testing. In contrast to non-fasting triglycerides, postprandial triglyceride evaluation refers to hourly triglyceride measurement following a predetermined high-fat meal in a laboratory setting. Generally, individuals are asked to report to a research laboratory after an overnight fast, a fasting blood draw is collected and then a standardised high-fat meal (i.e. either a set bolus of fat or a high-fat meal scaled to body weight) is consumed followed by hourly triglyceride measurement for 6–8 h. Other relevant modifiers of triglycerides such as physical activity and length of the overnight fast are also controlled. Although postprandial testing is ideal for determining an individuals’ peak triglycerides after a meal challenge and captures the total triglyceride area under the curve, postprandial protocols in their current form are time-intensive and not amenable to large-scale studies or clinical practice. Nonetheless, post-meal triglycerides, in particular postprandial triglycerides, appear to be a valuable marker for detecting early disease risk, warranting more efforts to make postprandial testing more clinically feasible.

In this review, we discuss commonalities and draw clear distinctions between the underlying physiological and metabolic changes that lead to elevated fasting, non-fasting and postprandial triglycerides and the implications of these changes for different triglyceride assessment methods. We also attempt to estimate the daily triglyceride burden experienced by the average individual consuming a Western-style diet, and review key pathophysiological mechanisms by which elevated fasting and non-fasting/postprandial triglycerides likely contribute to CVD risk. Lastly, we argue that in a subset of the population, postprandial triglycerides may be the preferred form of testing, even over non-fasting triglycerides, and offer suggestions to make postprandial protocols more practical.

## General overview of triglyceride metabolism

Triglycerides provide approximately 95 % of kilocalories derived from dietary fat and act as a stored energy reserve in adipose tissue. Due to their hydrophobic nature, triglycerides are transported within lipoproteins in the bloodstream along with cholesteryl ester, phospholipid and other fat-soluble molecules. In the fasted state, triglycerides are predominantly transported within apoB100 containing VLDL secreted by the liver^(1)^. Some triglyceride is also present in intermediate-density lipoproteins and LDL, which form as VLDL triglycerides are progressively cleaved by lipoprotein lipase (LPL), releasing free fatty acids to be utilised as energy or stored. Following a meal, it is normal to observe a moderate peak in triglycerides 3–5 h after eating, 80 % of which are packaged into apoB48 containing chylomicrons produced by enterocytes^(39)^. Chylomicrons initially enter the lymphatic system and ultimately reach the bloodstream where their triglyceride is also hydrolysed by LPL^(39)^. However, despite dietary triglyceride being mostly carried in chylomicrons, the increase in apoB containing particles after a meal is largely explained by a rise in VLDL, as postprandial VLDL secretion is only partially suppressed by insulin (approximately 50 %), and chylomicrons are preferentially hydrolysed by LPL^(34,40,41)^. Collectively, VLDL and chylomicrons are referred to as TRLs, and following LPL hydrolysis are termed TRL remnants. The dynamic process of TRL hydrolysis is influenced by many apolipoproteins that either activate LPL (e.g. apoC-II) or inhibit LPL such as apoC-I, apoC-III, angiopoietin-like protein-3, -4 and -8^(1,42–44)^. After the majority of TRL-triglyceride has been hydrolysed (approximately 6–8 h after a meal), TRL remnants and LDL are cleared by hepatic receptors (i.e. LDL receptor, LDL receptor-related protein-1), which is facilitated by apoE^(45–47)^.

## Estimated daily triglyceride kinetics in Westernised countries

**Fig. 2.**
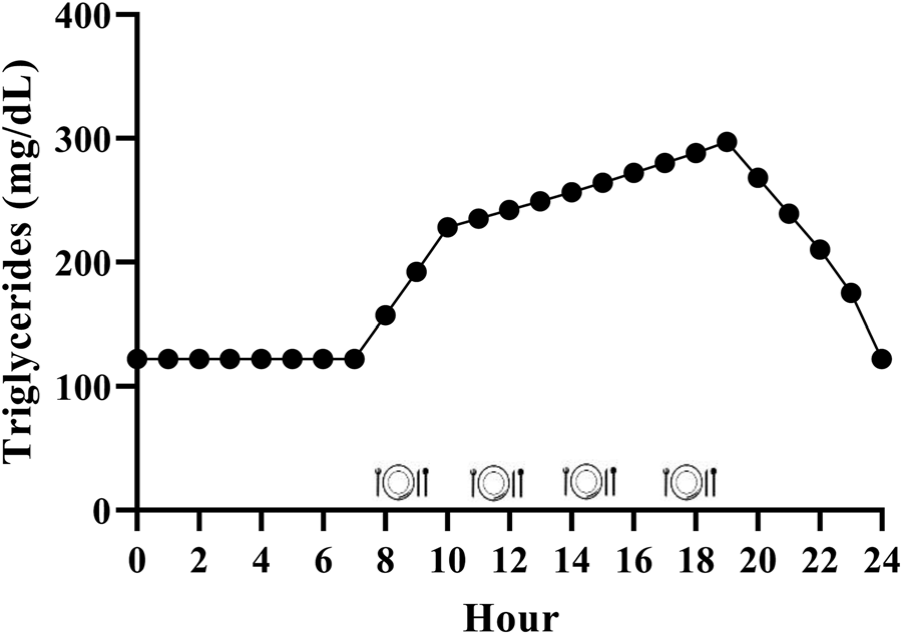
Estimated triglyceride kinetics of a typical U.S. male following a western dietary pattern. Since dietary triglycerides following a meal peak over 3–5 h, there is an additive effect of meals resulting in postprandial lipaemia during most of the day. Hour 0 represents midnight.

While it is generally accepted that triglycerides remain elevated for the majority of the day due to the additive effective of multiple meals, the exact magnitude is relatively unclear in the context of the typical Western dietary pattern. We have performed calculations to estimate this, and it appears that consuming the Western dietary pattern may lead to a substantial triglyceride peak, with triglycerides remaining above fasting 75 % of the day (Fig. 2). Consider the average American male whose fasting triglycerides are 1⋅37 mmol/l (122 mg/dl)^(1)^ and who consumes approximately 2400 kcal/d^(48)^, 35 % of which are from fat^(49)^ over 4–5 meals and snacks. These averages equate to consumption of approximately 93 g of fat each day, or approximately 23 g per meal if divided across four meals. Taguchi and colleagues^(50)^ determined that a similar quantity of fat (20 g) increased postprandial triglycerides by approximately 1⋅20 mmol/l (approximately 106 mg/dl) in subjects with normal fasting triglycerides <1⋅70 mmol/l (<150 mg/dl). Emerson *et al.* ^(26)^ demonstrated that consuming one moderate fat meal (sausage, egg, cheese, whole-grain crust; 8⋅5 kcal/kg; 30 % fat (13 % saturated fat)) containing similar total fat (22 g fat; 660 kcal), followed by the same meal 3 h later, sustained the initial triglyceride peak and caused approximately 3 % change per hour increase in serum triglycerides over the next 3 h before triglycerides began to decline. If four meals spaced 3 h apart are assumed, and postprandial triglyceride kinetics are similar following the third and fourth meals, we estimate that the average man may experience a triglyceride peak near 3⋅39 mmol/l (300 mg/dl) approximately 7 PM and will not return to baseline until approximately 1 AM (Fig. 2). Although this is a relatively crude estimate, specific meal patterns are assumed, and one would expect some differences in females due to the influence of sex hormones on triglycerides and having less visceral fat than men (presumably reducing hepatic VLDL-triglyceride secretion), it still likely provides insight into the daily triglyceride kinetics of many adults following a Western dietary pattern.

## Statistical considerations and epidemiology of fasting, non-fasting and postprandial triglycerides

### Statistical adjustments for fasting triglycerides

Although fasting triglycerides are the traditional method of triglyceride assessment, there has been extensive debate as to whether they are an independent risk factor for CVD. This controversy is largely based on whether other risk factors should be accounted for when evaluating the relationship between triglycerides and disease risk, implying that triglycerides are a correlate with metabolic disease rather than a causal factor. In most early studies where statistical adjustments were minimal, nearly all studies found that triglycerides were an independent CVD risk factor^(51)^. However, when adjustments for other factors, such as total cholesterol, HDL-C and BMI, were made, the association between fasting triglycerides and CVD was lost in some cases^(51)^. While this approach is logical, it should be considered that lipid concentrations often correlate with one another, an important example being the inverse relationship between triglycerides and HDL-C^(52)^. Presently, adjusting for these risk factors and others (e.g. blood pressure, smoking status) in large epidemiological cohorts is performed when examining the relationship between triglycerides and CVD in an attempt to tease apart the most relevant risk factors in multivariate analysis.

### Epidemiology of fasting triglycerides and CVD

In 1996, Hokanson and Austin^(53)^ performed a meta-analysis of seventeen prospective studies evaluating the relationship between fasting triglycerides and fatal and non-fatal cardiac events. Univariate analysis of >46 000 men revealed that a 1⋅00 mmol (89 mg/dl) increase in fasting triglycerides was associated with a 32 % increased relative risk of incident cardiac events. Similarly, women displayed a 76 % increased relative risk for cardiovascular events in a sample of nearly 11 000 women with a 1⋅00 mmol (89 mg/dl) increase in fasting triglycerides. After adjustment for age, total cholesterol, HDL-C and LDL-C, smoking, BMI and blood pressure, these relationships were still significant, but reduced to 14 and 37 % increased risk for men and women, respectively. Therefore, this large meta-analysis observed that fasting triglycerides were independently associated with cardiac events in men and women, although this relationship was weakened, but not completely negated, when considering other risk factors.

### Statistical adjustments for non-fasting triglycerides

Statistical adjustments made in studies evaluating non-fasting triglycerides and CVD risk largely include the same metabolic parameters as fasting triglycerides (e.g. other lipids, smoking, BMI). It is ideal to additionally adjust for meal timing and composition and physical activity, given that these factors have a large influence on non-fasting triglycerides and that non-fasting triglycerides can be measured within a large time frame and thus dramatically different results can be obtained depending on when the measurement is taken^(54)^. However, this data is frequently not available in large epidemiological cohorts, and this lack of control is the primary limitation of non-fasting triglyceride assessment.

### Epidemiology of non-fasting triglycerides and CVD

Several epidemiological studies have investigated the association between non-fasting triglycerides and CVD. In the Women's Health Study cohort (26 000 initially healthy women, mean follow-up of 11⋅4 years), those that fell within the top two tertiles of non-fasting triglycerides (i.e. 1⋅19–1⋅21 mmol/l (105–107 mg/dl) and ≥1⋅93 mmol/l (171 mg/dl)) were at 44 and 98 % increased risk for incident CVD in the fully adjusted model, which accounted for total and HDL-C, C-reactive protein and BMI, among other covariates^(22)^. In contrast, the association between fasting triglycerides and CVD was lost in the fully adjusted model, suggesting that non-fasting triglycerides may have more utility than fasting triglycerides for predicting future CVD events. When reanalysing the data as quintiles, only the highest quintile of non-fasting triglycerides (≥ 2⋅43 mmol/l or 215 mg/dl) was associated with increased risk for CVD events (99 % increased relative risk) after adjustments for covariates, and again, there was no association with fasting triglycerides.

Similarly, the Copenhagen City Heart Study (approximately 14 000 men and women; mean follow-up >26 years) found that incidence of myocardial infarction, ischaemic heart disease and total mortality significantly increased within all five quintiles of non-fasting triglycerides, beginning as low as 1⋅00–1⋅99 mmol/l (89–176 mg/dl), compared with the reference group (<1⋅00 mmol or 89 mg/dl) in both men and women^(21)^. Additionally, fully adjusted hazard ratios revealed that for every 1⋅00 mmol/l (89 mg/dl) increase in non-fasting triglycerides, women were 20 and 18 % more likely to experience myocardial infarction and death, respectively. However, in men, no relationship was observed between cardiac events or mortality and non-fasting triglycerides in the full model, despite 6–10 % increased risk for myocardial infarction, ischaemic heart disease and mortality in models adjusting only for age and HDL-C.

In the Norwegian Counties Study (86 000 men and women; mean follow-up 27 years), non-fasting triglycerides were associated with increased all-cause mortality and death from CVD in women within the main study population following adjustment for typical covariates (but not HDL-C), which included time since last meal^(20)^. Women were at 17 % increased risk of all-cause mortality beginning at 1⋅02 mmol/l (90⋅3 mg/dl) of non-fasting triglycerides with the highest quintile (>1⋅71 mmol/l; >151⋅5 mg/dl) exhibiting 42 % increased risk. Furthermore, women in the second, third and fourth quintiles were 28–37 % more likely to die of CVD relative to the lowest quintile, and the highest quintile was associated with 77 % increased risk of CVD death. In men, these relationships were generally not observed, although a 20–21 % increased risk of death from ischaemic heart disease was seen in the two highest quintiles of non-fasting triglycerides. Interestingly, in a subset where HDL-C data was available and included in the model, for every 1 mmol/l (89 mg/dl) increase in non-fasting triglycerides, a 6 % increase risk of all-cause mortality was observed in both sexes, as well as a 6 % increased risk of death from CVD in men. Taken together, epidemiological evidence suggests that non-fasting triglycerides are associated with CVD events and death, even after adjustment for relevant confounding variables, and are arguably a better predictor of CVD than fasting triglycerides in some populations.

### Statistical adjustments for postprandial triglycerides

While many of the same general and metabolic variables (e.g. age, sex, BMI, cholesterol metrics) are also often adjusted for in small postprandial studies when sample size permits, the limitations and ideal statistical adjustments relevant to non-fasting triglycerides are directly accounted for in the postprandial study design. Specifically, participants are typically asked to refrain from exercise before and during the postprandial fat tolerance test and the time since their last meal is either controlled for or recorded by study personnel by virtue of study design, removing the need for statistical adjustment. However, this level of control compromises the size of the study population, limiting the number statistical adjustments that can be made.

### Epidemiology of postprandial triglycerides and CVD

To our knowledge, only one cohort study has evaluated the relationship between postprandial triglycerides determined by a controlled fat tolerance test and CVD outcomes^(55)^. The Atherosclerosis Risk in Communities (ARIC) study administered a fat tolerance test to 559 participants and then assessed the relationship between postprandial lipaemia and CVD events over a 20-year follow-up. The liquid test meal utilised was a set bolus and composed of heavy whipping cream, ice cream, safflower oil, chocolate syrup and protein powder (1265 kcal; 105 g fat (50 % saturated fat)). Among several indicators of postprandial lipaemia (i.e. triglycerides, TRL-triglycerides, retinyl palmitate and the apoB48/apoB100 ratio), there was no relationship between postprandial lipaemia and CVD events when the population was divided into tertiles. Although this was an important first attempt to study postprandial triglycerides and CVD outcomes in an epidemiological context, the investigators acknowledged that this study was underpowered and larger-scale studies similar in study design are needed.

## Genetic evidence for involvement of triglycerides in CVD

While epidemiological evidence is somewhat conflicting, genetic evidence provides another perspective on the role of triglycerides in CVD risk. That is, genetic studies consistently support some involvement of triglycerides and/or their clearance in the atherosclerotic process. For example, both rare loss-of-function mutations and more common variants in the *LPL* gene lead to elevated triglycerides and are associated with increased CVD risk^(56,57)^. Similarly, variants in the *APOA5* gene, another apolipoprotein involved in LPL activation, selectively increase triglycerides and the likelihood of developing CVD^(58,59)^. Conversely, loss-of-function mutations in genes encoding LPL inhibitors (i.e. *APOC3*, *ANGPTL4*) are associated with reduced triglycerides and lower risk of CVD^(60,61)^. Using genetic risk scores, Ference and colleagues^(62)^ compared the risk reduction associated with lowering triglycerides and LDL-C. Interestingly, the effect of lowering triglycerides and LDL-C normalised to apoB particles were both associated with an approximately 33 % reduced risk for CVD, suggesting that there may be a benefit to lowering triglycerides specifically beyond what fibrates can generally accomplish. This genetic benefit from lowering triglycerides was attributed to a reduction in VLDL particles, which are arguably more atherogenic than LDL, albeit fewer in number^(62,63)^. Overall, these studies demonstrate that genetic mutations leading to reduced triglycerides (regardless of fasting or non-fasting context) reduce the risk for CVD and imply that there may be a cardiovascular benefit to lowering fasting and non-fasting/postprandial triglycerides.

## Mechanisms and pathophysiological relevance of high fasting triglycerides

### Mechanisms leading to high fasting triglycerides

**Fig. 3.**
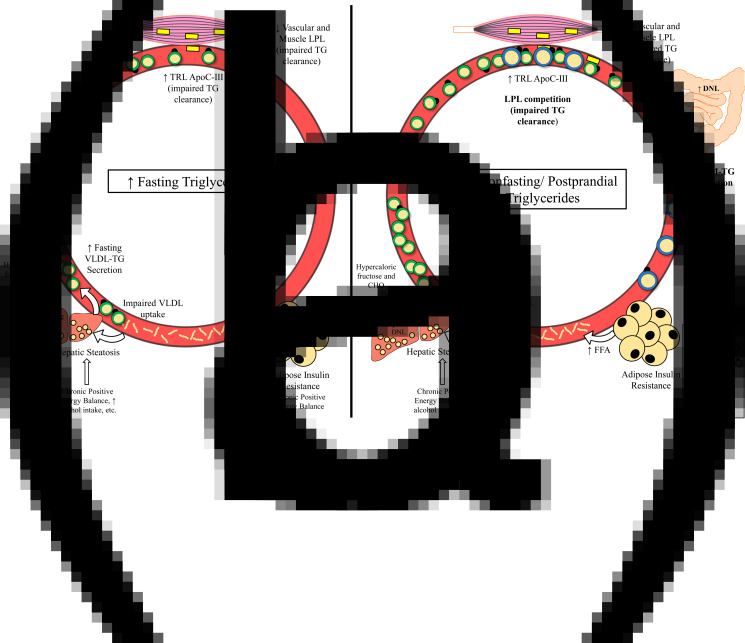
Mechanisms leading to raised fasting *v*. non-fasting/postprandial triglycerides. (a) Mechanisms leading to high fasting triglycerides. Primary drivers of high fasting triglycerides appear to be hepatic steatosis (driven in part by adipose insulin resistance and lifestyle factors) and subsequent increased VLDL-triglyceride secretion. Impaired triglyceride clearance due to reduced LPL activity and enrichment of TRLs with apoC-III are also implicated in high fasting triglycerides. (b) Mechanisms leading to high non-fasting/postprandial triglycerides. In addition to mechanisms that also increase fasting triglycerides, failure of insulin to suppress postprandial VLDL secretion, competition between VLDL-triglycerides and chylomicron-triglycerides for LPL hydrolysis, and oversecretion of intestinal chylomicrons are unique drivers of high non-fasting/postprandial triglycerides. *Abbreviations*: apo, apolipoprotein; CHO, carbohydrate; CM, chylomicron; DNL, *de novo* lipogenesis; FFA, free fatty acid; LPL, lipoprotein lipase; TG, triglyceride; TRL, triglyceride-rich lipoprotein; VLDL, very-low-density lipoprotein.

Given that there is reason to believe that triglycerides and/or post-meal triglycerides are involved in the atherosclerotic process, it is important to understand the underlying causes and potential pathophysiological relevance of elevated fasting, non-fasting and postprandial triglycerides. High fasting triglycerides are generally the result of increased VLDL-triglyceride secretion (commonly related to hepatic steatosis) and/or impaired triglyceride clearance by LPL^(64)^ (Fig. 3(a)). These metabolic changes leading to elevated fasting triglycerides have been attributed to many factors including insulin resistance, diet and lifestyle behaviours that promote obesity and ectopic fat accumulation^(7–12,65,70,71,84,85)^ (Table 1). Perhaps the most accepted mechanism for increasing fasting triglycerides is adipose tissue insulin resistance, leading to uninhibited lipolysis, increased free fatty acid flux to the liver and ultimately increased VLDL-triglyceride secretion^(7–9,129)^. Indeed, the homeostatic model of insulin resistance (HOMA-IR) strongly correlates with triglyceride-rich VLDL_1_ secretion and VLDL_1_ apoB pool size^(12)^. While visceral adipose tissue is more insulin resistant than subcutaneous fat, and consequently is often cited as a contributor to elevated triglycerides, it should be noted that the majority of free fatty acids reaching the liver are derived from peripheral depots^(130,131)^. Lastly, enhanced hepatic *de novo* lipogenesis in the fasted state appears to contribute to liver fat accumulation and subsequent VLDL secretion in obese, hyperinsulinemic individuals^(65)^.

**Table 1.** Non-genetic factors contributing to elevated fasting and non-fasting/postprandial triglycerides

Physiological disturbance	Pathophysiological mechanism leading to hypertriglyceridemia	Reference
Insulin resistance-related		
Adipose insulin resistance	↑ lipolysis and FFA flux to liver → ↑ VLDL secretion	^(7–9)^
Hepatic insulin resistance	↑ VLDL secretion	^(10–12)^
	↑ DNL	^(14,65,66)^
	↓ **suppression of postprandial VLDL**	^(**67,68**)^
Reduced LPL activity	Delayed VLDL clearance in fasted state	^(31–33)^
	**Delayed CM and VLDL clearance postprandially**	^(**31–33**)^
	↑ apoC-III → LPL inhibition, impaired TRL clearance	^(69)^
**Intestinal CM over secretion**	↑ **apoB48 stability, intestinal DNL and MTP stability**	^(**28–30**)^
Diet-related		
High-carbohydrate diets (>60 %)	↑ DNL	^(65,70,71)^
High fructose intake (chronic)	↑ visceral adipose, hepatic insulin resistance, DNL → ↑ VLDL	^(72–78)^
**High fructose intake (acute)**	↑ **DNL**	^(**72,79,80**)^
Chronic positive energy balance	↑ total, visceral and hepatic fat → ↑ VLDL production, insulin resistance	^(81–83)^
High alcohol consumption (chronic)	↑ in VLDL secretion, inhibition of LPL, ↑ adipose lipolysis and VAT	^(84–87)^
**High alcohol consumption (acute)**	↑ **in VLDL secretion, inhibition of LPL**	^(**88–90**)^
**High-fat meal rich in TGs (acute)**	↑ **CM secretion**	^(**1**)^
**LPL saturation**	**Competition between high postprandial CM and VLDL**	^(**1**)^
Disease-related		
Hypothyroidism	↓ LPL activity	^(91–93)^
	↓ LDL uptake (contain some TG)	^(93–95)^
Uncontrolled T1D	↑ VLDL production	^(96–98)^
	↓ adipose LPL activity	^(31,99,100)^
T2D	↑ VLDL production	^(4–6)^
	↓ CM clearance	^(101)^
	↓ activity/quantity of LPL	^(31–33)^
	↑ apoC-III: apoC-II ratio → favours LPL inhibition, impaired TRL clearance	^(102)^
Obesity	↓ adipose LPL and LRP1 gene expression	^(103)^
	↑ FFA influx to liver → ↑ VLDL secretion	^(1,15,16)^
	↑ CETP activation → ↑ transfer of TGs to HDL and LDL	^(104)^
Hepatic steatosis/NAFLD	↑ DNL and TG storage → ↑ VLDL secretion	^(13,14)^
Chronic kidney disease	↓ LPL activity	^(105,106)^
	↓ apoC-II → ↓ LPL activation	^(107)^
	↑ apoC-III → LPL inhibition, impaired TRL clearance	^(106,107)^
Lifestyle-related		
Increased visceral adipose	↑ FFA influx to liver → ↑ VLDL secretion	^(1,15,16)^
Low muscle mass	↓ quantity of muscle LPL	^(108)^
Physical inactivity	Lack of physical activity induced muscle LPL quantity/activity	^(109–111)^
Smoking	Potentially inflammation and insulin resistance	^(112–114)^
Other		
Low blood plasma volume	↑ circulating concentration of TGs	^(115–117)^
Age	Likely multifactorial including ↑ visceral adipose, physical inactivity, etc.	^(118)^
Sex	Women display greater apoC-III/C-II ratio than men → favours LPL inhibition, impaired TRL clearance	^(119)^
Oral contraceptive use	↓ LPL activity, increased VLDL secretion	^(120–122)^
Pregnancy	↑ adipose lipolysis → ↑ VLDL secretion, reduced adipose LPL activity	^(123–125)^
Menopause status	↑ visceral fat → ↑ risk for insulin resistance	^(126–128)^

*Notes*: Potential factors other than genetic lipid disorders leading to elevated fasting and non-fasting/postprandial triglycerides. All factors described can contribute to hypertriglyceridemia, but many cluster together and are likely interrelated. Plain text indicates factors that contribute to high fasting triglycerides and by extension also partially contribute to non-fasting/postprandial triglycerides. Highlighted and bolded rows denote factors uniquely leading to elevated non-fasting/postprandial triglycerides.

*Abbreviations*: apo, apolipoprotein; CETP, cholesterol ester transfer protein; CM, chylomicrons; DNL, *de novo* lipogenesis; FFA, free fatty acid; HDL, high-density lipoprotein; LDL, low-density lipoprotein; LPL, lipoprotein lipase; LRP1, LDL receptor-related protein-1; MTP, microsomal triglyceride transfer protein; T1D, type 1 diabetes; T2D, type 2 diabetes; TG, triglycerides; TRL, triglyceride-rich lipoprotein; VAT, visceral adipose tissue; VLDL, very-low-density lipoprotein.

From a triglyceride clearance standpoint, insulin resistance and type 2 diabetes are associated with reduced adipose *LPL* expression and LPL activity, interfering with hydrolysis of VLDL-triglyceride and likely prolonging VLDL particle residence time^(31–33,132)^. Insulin resistance is also characterised by higher serum apoC-III, which contributes to high triglycerides by inhibiting apoE mediated uptake of apoB particles and reducing triglyceride hydrolysis by LPL^(69)^.

### Pathophysiological relevance of high fasting triglycerides

Despite the controversy over whether triglycerides are an independent risk factor for CVD, mechanistic work provides indirect explanations by which chronically elevated fasting triglycerides are a cardiometabolic liability. In the context of insulin resistance, triglyceride-enriched VLDL are oversecreted^(10–12)^ and cleared more slowly than in insulin-sensitive individuals^(31–33)^. Due to longer residence time and insulin's activation of cholesterol ester transfer protein (CETP), triglyceride in VLDL is transferred to HDL and LDL particles to a greater degree^(1)^. After triglyceride is further hydrolysed from these HDL and LDL by hepatic lipase, they adopt a small, dense phenotype. Consequences of these small, dense LDL and HDL include impaired LDL uptake by the LDL receptor, higher likelihood LDL are retained in the subintimal space, and increased HDL degradation leading to decreased circulating HDL. Together, these adverse lipid changes are associated with increased CVD risk and can be traced back to a high hepatic triglyceride burden and VLDL secretion^(1)^.

Another potential indirect role of disturbed triglyceride metabolism in the fasted state is a higher burden of VLDL remnants, which are rich in cholesterol and have similar properties to pro-atherogenic LDL. That is, remnants <70 nm in diameter can enter the subintimal space, are selectively retained, and may be phagocytosed by macrophages forming foam cells in their unoxidised form, thereby contributing to atherosclerotic plaque^(36,133,134)^.

*In vitro* work has demonstrated TRL remnants and TRL lipolysis products have pro-inflammatory properties that may promote CVD. Specifically, TRL remnants increase monocyte adhesion to endothelial cells by upregulating protein expression of adhesion molecules (i.e. vascular cell adhesion molecule (VCAM)-1, intracellular adhesion molecule (ICAM)-1, E-selectin), increase endothelial production of tumour necrosis factor (TNF)-α and interleukin (IL)-1β, and promote platelet activation^(135–137)^. These effects of remnants may be related to TRL enrichment of apoC-III, which is common in dyslipidemic individuals and similarly activates NF-κB and increases monocyte adhesion^(138–140)^ and/or production of triglyceride lipolysis products by LPL which may promote inflammation and endothelial cell apoptosis^(141–144)^.

Remnant TRLs and their high cholesterol content, rather than the triglycerides they carry, seem to be primarily responsible for the adverse effects associated with high triglycerides. However, it cannot be ignored that VLDL-triglyceride export is generally viewed as driven by increased substrate (i.e. triglyceride, fatty acids) reaching the liver^(130)^. Therefore, regardless of the source of hepatic triglyceride (i.e. hepatic uptake of free fatty acids, *de novo* lipogenesis or previously accumulated hepatic triglyceride), the more VLDL that will be secreted and the more remnants that will be in circulation. Overall, high fasting triglycerides appear to indirectly promote LDL and VLDL remnant retention in the subintimal space, reduce HDL, and potentially increase inflammation and endothelial cell dysfunction.

## Mechanisms and pathophysiological relevance of high non-fasting/postprandial triglycerides

### Mechanisms leading to high non-fasting/postprandial triglycerides

Since the difference between non-fasting and postprandial triglycerides is related to assessment method and not the underlying physiology, we will not distinguish between these terms in this section. Several mechanisms exist that uniquely contribute to high triglycerides in the fed state, many of which are tied to insulin resistance (Fig. 3(b)). Perhaps the most intuitive pathway is the competition of newly secreted chylomicron-triglyceride and already present VLDL-triglyceride for LPL hydrolysis^(1,31–33)^. This phenomenon occurs in both healthy and insulin-resistant individuals; however, in the context of insulin resistance, VLDL secretion is poorly suppressed and LPL activity is reduced, promoting an even greater and sustained postprandial response^(31–33,67,68,101)^. Further exacerbating competition for LPL hydrolysis is that intestinal chylomicrons are oversecreted in insulin-resistant individuals, which appears to be due to increased stability of proteins needed for chylomicron formation (i.e. apoB48, microsomal transfer protein) and enhanced *de novo* lipogenesis within enterocytes^(28–30)^. Importantly, increased chylomicron secretion and subsequent uptake of chylomicron remnants by the liver could be another mechanism contributing to hepatic steatosis, potentially exacerbating postprandial dyslipidemia in the future^(130)^. Postprandial hepatic *de novo* lipogenesis was reported to be approximately 14 and 18 % greater relative to the fasted state after consumption of two consecutive high-carbohydrate mixed meals (54 % CHO, 32 % fat, 14 % pro), contributing to the postprandial triglyceride pool^(145)^. Although fasting triglycerides are a strong predictor of peak postprandial triglycerides, these mechanisms may explain why some have a disproportionately high triglyceride response following a meal. Furthermore, one or more of these processes are likely occurring in those who have elevated non-fasting/postprandial triglycerides^(23,146)^.

### Pathophysiological relevance of high non-fasting/postprandial triglycerides

Although mechanisms involving CETP-mediated lipid exchange are relevant in the postprandial state, much attention has been paid to TRL remnants as a major pathophysiological mechanism linking postprandial lipaemia to increased CVD risk. TRL remnants are far more numerous in the fed state due to the addition of intestinal TRLs and the temporary delayed VLDL hydrolysis due to chylomicron competition and LPL saturation. Given that high-fat meals lead to increased chylomicron secretion^(147)^, increasing dietary triglyceride will increase the number of chylomicron remnants with pro-atherogenic potential and will delay hydrolysis of existing VLDL/VLDL remnants by LPL. In the context of very pronounced postprandial lipaemia, whether diet- and/or metabolic disease-induced, remnants will have even longer residence time, increasing their likelihood of entering the subendothelial space.

High-fat meals are associated with several other pro-atherogenic changes within the postprandial period. From an inflammatory standpoint, IL-6, IL-8, TNF-α and adhesion molecules (i.e. sICAM-1, sVCAM-1) are increased in the serum following a high-fat meal^(148–150)^. In the case of Nappo *et al.*^(150)^, several of these inflammatory markers (i.e. TNF-α, IL-6 and VCAM-1) correlated with postprandial triglycerides. Similar to studies discussed with fasting triglycerides, mechanistic evidence suggests that increased postprandial inflammation may be linked to TRLs and their triglyceride lipolysis products. In an endothelial cell line treated with TRLs from hypertriglyceridemic subjects, both fasting and postprandial TRLs upregulated genes encoding adhesion molecules such as VCAM-1, E-selectin and platelet endothelial cell adhesion molecule 1 (PECAM-1), among others^(151)^. Importantly, for many of these genes, the response was even greater when postprandial TRLs were used relative to fasting TRLs. A similar study suggested postprandial, but not fasting, TRLs increased adhesion molecule expression and low-dose TNF-α must be present^(152)^. Fasting and postprandial TRLs similarly increased NF-κB DNA binding activity more than 2-fold *in vitro*, which in theory could lead to increased transcription of a host of inflammatory mediators. Taken together, fasting and postprandial TRLs may increase inflammation through similar mechanisms, but there is reason to believe that this effect is more pronounced with postprandial TRLs.

Inflammation resulting from high-fat meals, and potentially TRLs, appears to be largely explained by innate immune system activation. For example, after a fat bolus, circulating neutrophils increased by 59 % from baseline and peaked alongside postprandial triglycerides^(153)^. Furthermore, increased serum lipopolysaccharide (LPS) and indicators of the LPS-induced innate immune response (i.e. LPS binding protein and peripheral blood mononuclear cell of toll-like receptor (TLR)-4 protein abundance) are observed postprandially^(154,155)^. Traditionally, the increase in LPS from the gut and subsequent TLR-4 signalling has been considered a primary driver of postprandial inflammation. However, a recent report suggested that high-fat meal induced postprandial inflammation may be more related to hydrolysis of TRL-triglyceride by LPL than LPS^(156)^. This finding is consistent with work showing that the natural lipolysis of TRL-derived saturated free fatty acids (i.e. lauric acid) promotes a TLR-4 and TLR-2-mediated immune response in monocyte/macrophage cell lines^(141–143)^. Complement component 3 (C3) rises following a fat bolus in both healthy controls and individuals with coronary artery disease^(157)^. *In vitro* evidence suggests that this phenomenon may be due to the interaction between chylomicrons and adipocytes, which prompts adipocytes to release C3^(158)^. However, protein expression of the classical complement pathway inhibitor C4b-binding protein is increased in the postprandial period, partially bringing into question the relevance of increased postprandial C3^(159)^.

In addition to inflammation, several other adverse effects have been associated with postprandial lipaemia. It is well-established that vascular function (as measured by flow-mediated dilation) predicts future CVD and is impaired following a high-fat meal^(160,161)^. The negative effect of high-fat meals on vascular function may be due to increased reactive oxygen species, which both decrease nitric oxide production and reduce nitric oxide bioavailability^(160,162)^. Indeed, many reports indicate that postprandial triglycerides, flow-mediated dilation, and/or markers of oxidative stress correlate with one another. However, these relationships are not observed universally and can be seen after a glucose bolus and mixed meals as well^(153)^. Regardless of the instigating meal, postprandial oxidative stress likely accounts for elevated postprandial oxidised LDL^(163,164)^, which could enhance foam cell formation. Lastly, the angiogenic factors vascular endothelial growth factor (VEGF)-A and VEGF-C increased in response to a high-fat meal, but implications of this are unclear as VEGF promotes both blood vessel growth and is implicated in plaque expansion and potentially haemorrhage^(149,165,166)^.

## Clinical implementation

While there is no clear consensus, the evidence discussed thus far suggests at minimum an indirect role of triglycerides in the atherosclerotic process, making it important to identify the best method for triglyceride assessment. In many countries, triglycerides are still largely measured and interpreted in the fasted state to avoid the influence of diet and other confounders that accompany waking hours (e.g. physical activity, meal timing). As a result, fasting triglycerides have been extensively studied and have a well-established reference range of <1⋅70 mmol/l (150 mg/dl). Another major advantage with fasting triglycerides is that only one measurement is required and results can be generated in minutes. Despite the advantages of fasting triglycerides as a screening tool, they are less predictive of disease, and appear to be less sensitive than non-fasting and postprandial triglycerides for detecting CVD risk.

**Fig. 4.**
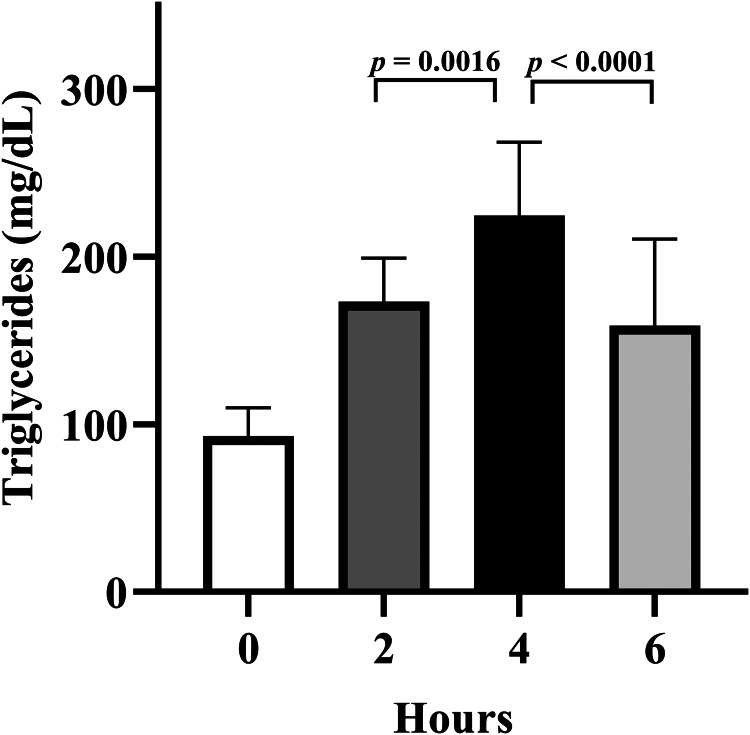
Adverse postprandial triglyceride response may not be detected with non-fasting triglyceride measurement. Individuals who presented with normal fasting triglycerides (<1⋅70 mmol/l or 150 mg/dl), yet experienced an adverse postprandial response (i.e. ≥2⋅26 mmol/l or 220 mg/dl) after being challenged with a high-fat meal (10–13 kcal/kg body mass; 61–64 % kcal from fat) were pooled from several studies (*n* 17). Paired *t*-tests were utilised to evaluate the difference between 2 *v*. 4 h and 4 *v*. 6 h. Despite this group having an adverse postprandial response, on average, they would not meet criteria for an adverse non-fasting triglyceride response at both 2 and 6 h, which are both acceptable times for non-fasting triglyceride measurement.

Non-fasting triglycerides include triglyceride measurements taken within 8 h of eating and are also easily measured with one blood draw. Non-fasting triglyceride measurement has not traditionally been the first choice of measurement in most countries, but several bodies including the American Heart Association, European Atherosclerosis Society, Danish Society for Clinical Chemistry, among others, now recommend non-fasting lipid panels be performed^(1,24,167)^. Although an updated 2019 expert panel report recommends postprandial testing when non-fasting triglycerides are 1⋅30–2⋅26 mmol/l (115–200 mg/dl), European bodies and the American Heart association cut points are not as aggressive, defining ideal non-fasting triglycerides as <1⋅98 mmol/l (175 mg/dl) and <2⋅26 mmol/l (200 mg/dl), respectively^(1,24)^. However, data from our lab suggests that individuals who have an adverse postprandial response following a fat tolerance test may be overlooked using these non-fasting reference ranges. In individuals with normal fasting triglycerides (<1⋅70 mmol/l or 150 mg/dl) subjected to a fat tolerance test who go on to have an adverse postprandial triglyceride response (≥2⋅26 mmol/l or 220 mg/dl), their mean 2-h and 6-h triglycerides are below the non-fasting recommendations of 1⋅98 and 2⋅26 mmol/l (175 and 200 mg/dl), and significantly lower than peak 4-h triglycerides (Fig. 4). Since 2 and 6 h post-meal is well within the 8-h non-fasting assessment window, and the amount of fat consumed prior to a non-fasting blood draw would often be less than in these postprandial lipid challenges, these at-risk individuals may be overlooked based on non-fasting triglycerides, but their CVD risk would be realised if subjected to a standardised fat tolerance test.

Postprandial triglycerides are almost exclusively measured in a research laboratory setting over periods of 6–8 h post-meal consumption with serial blood draws (often hourly). With this design, virtually all limitations of non-fasting triglycerides are addressed. Specifically, all postprandial studies control for meal composition, quantity and timing, and for exercise prior to and during the test. All of these factors make postprandial triglyceride assessment a more robust and insightful clinical measurement than non-fasting triglycerides, while still maintaining sensitivity. Unfortunately, the rigour associated with postprandial testing is expensive and time-consuming for both researchers and participants, hindering widespread adoption. In order to address these issues, ideally one, simplified fat tolerance test would be adopted by all. Our group has devoted work to this issue with some success. We recently determined that an abbreviated and simplified fat tolerance test was both valid and reliable^(168,169)^. With this protocol, blood draws only occur at baseline and 4 h, and participants are allowed to leave the laboratory and engage in normal daily activities as long as they do not eat or perform planned exercise. This abbreviated postprandial protocol, or something similar, could be utilised in research and clinical settings for determination of postprandial lipaemia in a widespread manner.

One barrier to clinical implementation of postprandial triglyceride measurement is the absence of a standardised test meal. Across the postprandial literature, substantial variability exists in the composition of high-fat meals used to test postprandial lipaemia. In our abbreviated fat tolerance test mentioned earlier, the test meal is composed of coconut cream, chocolate syrup and protein powder (9 kcal/kg; 73 % fat (86 % saturated fat)), which allows for individuals with food allergies and/or following a vegan diet to consume it. This generally aligns well with a recommendation for oral fat tolerance testing from an expert panel^(24)^, which recommended a set bolus of 75 g fat, 25 g carbohydrate and 10 g protein composed of whipped cream or cream cheese with added sugar. Overall, an agreed-upon standardised fat challenge is necessary before widespread implementation of postprandial triglyceride testing can be realised.

## Conclusion/future directions

While much attention has been paid to reducing triglycerides as a strategy for reducing residual risk of CVD after lowering LDL-C, there is growing support of a role for post-meal triglycerides, and perhaps postprandial measurements in particular, as a tool for screening cardiometabolic risk. Nonetheless, several questions have yet to be addressed. First, in light of the opposing epidemiological evidence between non-fasting triglycerides and postprandial triglycerides, a larger prospective study focused on postprandial triglycerides specifically is needed. Based on the results of this future study and others, universally accepted reference ranges for non-fasting and postprandial triglycerides are needed that correspond with CVD risk.

In sum, our understanding of triglycerides and CVD risk has evolved dramatically since the 1950s, including the realisation that non-fasting and postprandial triglycerides appear to be useful CVD risk factors. Although there are many other useful CVD risk factors (e.g. LDL-C, HDL-C, body composition, etc.), our data and others suggest that triglycerides, in particular postprandial triglycerides, are an independent, sensitive and useful screening tool for cardiometabolic health. Future work should attempt to identify those whose CVD risk is ambiguous and would most benefit from postprandial triglyceride testing.
